# The Dual Role of Cellular Senescence in Developing Tumors and Their Response to Cancer Therapy

**DOI:** 10.3389/fonc.2017.00278

**Published:** 2017-11-23

**Authors:** Markus Schosserer, Johannes Grillari, Michael Breitenbach

**Affiliations:** ^1^Department of Biotechnology, University of Natural Resources and Life Sciences, Vienna, Austria; ^2^Austrian Cluster for Tissue Regeneration, Vienna, Austria; ^3^Christian Doppler Laboratory for Biotechnology of Skin Aging, Vienna, Austria; ^4^Evercyte GmbH, Vienna, Austria; ^5^Department of Cell Biology, Division of Genetics, University of Salzburg, Salzburg, Austria

**Keywords:** aging, cancer, cellular senescence, cancer-associated fibroblasts, senescence-associated secretory phenotype, stress-induced premature senescence, senolytic compounds, modulation of protein synthesis

## Abstract

Cellular senescence describes an irreversible growth arrest characterized by distinct morphology, gene expression pattern, and secretory phenotype. The final or intermediate stages of senescence can be reached by different genetic mechanisms and in answer to different external and internal stresses. It has been maintained in the literature but never proven by clearcut experiments that the induction of senescence serves the evolutionary purpose of protecting the individual from development and growth of cancers. This hypothesis was recently scrutinized by new experiments and found to be partly true, but part of the gene activities now known to happen in senescence are also needed for cancer growth, leading to the view that senescence is a double-edged sword in cancer development. In current cancer therapy, cellular senescence is, on the one hand, intended to occur in tumor cells, as thereby the therapeutic outcome is improved, but might, on the other hand, also be induced unintentionally in non-tumor cells, causing inflammation, secondary tumors, and cancer relapse. Importantly, organismic aging leads to accumulation of senescent cells in tissues and organs of aged individuals. Senescent cells can occur transiently, e.g., during embryogenesis or during wound healing, with beneficial effects on tissue homeostasis and regeneration or accumulate chronically in tissues, which detrimentally affects the microenvironment by de- or transdifferentiation of senescent cells and their neighboring stromal cells, loss of tissue specific functionality, and induction of the senescence-associated secretory phenotype, an increased secretory profile consisting of pro-inflammatory and tissue remodeling factors. These factors shape their surroundings toward a pro-carcinogenic microenvironment, which fuels the development of aging-associated cancers together with the accumulation of mutations over time. We are presenting an overview of well-documented stress situations and signals, which induce senescence. Among them, oncogene-induced senescence and stress-induced premature senescence are prominent. New findings about the role of senescence in tumor biology are critically reviewed with respect to new suggestions for cancer therapy leveraging genetic and pharmacological methods to prevent senescence or to selectively kill senescent cells in tumors.

## Introduction

In current cancer research, the tumor microenvironment is coming more and more into the focus as it is able to either promote or inhibit carcinogenesis and metastasis by providing cancer cells with growth factors and supply of oxygen and nutrients. The stroma of tumors is enriched for chemokines, which attract and activate various other cell types, including cancer-associated fibroblasts (CAF). These cells closely interact with cancer cells, secrete cytokines, remodel the extracellular matrix and thereby promote malignancy (1). Importantly, age is one of the main risk factors for many types of cancer and is accompanied by an accumulation of senescent cells in various tissues of the body. As senescent cells actively shape their tissue microenvironment in a similar fashion as CAF toward a pro-tumorigenic state (2), cellular senescence (together with the well-known mutation accumulation over a lifetime) is probably one of the main contributors to age-associated cancer development.

Cellular senescence, a state of irreversible growth arrest, was discovered by Leonard Hayflick more than 50 years ago (3). A whole new field of investigation was opened up by this seminal discovery that was over the last 50 years closely intertwined with research in organismic aging, which was of obvious primary interest, but also with several other closely related fields, like oxidative stress research, origin of reactive oxygen species (ROS), role of mitochondria in aging, role of telomeres and telomerase in aging, and the genetics of stress response and stress defense. From early on in this field, the hypothesis was entertained that (i) the phenomenon observed in mammalian cell culture indeed occurs *in vivo* and drives normal organismic aging and (ii) induction of senescence was positively selected for in evolution for several reasons, among them to protect cells and organisms from cancer. Both of these ideas were highly speculative, but over the last 20 years were shown to be correct in part (2, 4–8). On the other hand, reports that establish a beneficial and important role of cellular senescence in embryogenesis (9, 10) and wound healing (11) imply that senescence might have evolved for other reasons as well.

The basic arguments about the role of senescence in cancer protection are as follows: senescent cells have lost the ability to undergo cell division permanently, although they may be metabolically fully active. This would certainly protect individuals carrying a primary cancer from further cancerous growth. However, this has to be seen in a different way nowadays as compared to the time when this “anticancer hypothesis” was first published (8), as knowledge of the genetics of cancer and senescence increased rapidly over the last few years. By this, we mean on the one hand the sequence of mutational events that takes place in growing tumors (12, 13), and on the other hand the knowledge of biochemical senescence markers in senescent cells *in vivo* (6, 14–17). Most importantly, senescent cells may be prone to genetic and epigenetic instability (18, 19), which is also a hallmark of cancer cells (12). In addition, the senescence-associated secretory phenotype (SASP) directly causes transformation of neighboring cells and destruction of the extracellular matrix, other hallmarks of cancer growth, which help to spread malignant cells in the body (2, 20, 21). Thus, cellular senescence can be viewed as a typical example for antagonistic pleiotropy: at young age, senescence might protect cells from transformation into primary tumors; however, at old age senescent cells generate a pro-tumorigenic microenvironment.

In this review, we will summarize mechanisms of senescence induction, especially in the context of aging-associated cancers and tumor therapy. While cellular senescence was originally believed to be caused by telomere shortening alone, increasing evidence suggested additional inducers of senescence. These inducers of senescence include the activation of DNA damage response pathways by cytotoxic compounds or ROS as well as activation of oncogenes. The contribution of senescent cells to a pro-oncogenic microenvironment will be discussed and compared to other cancer-associated cells, such as CAF. Finally, we will introduce current and future therapy options targeting cancer-, non-senescent-, and senescent cells and discuss their potential influence on cell fate decisions within the tumor stroma.

## Mechanisms of Cellular Senescence Induction and Their Connection with Cancer Biology

### Biomarkers of Cellular Senescence

For a long time, since the discovery of replicative senescence in cell culture (3) until relatively recently [summarized in Ref. (22)], it was not clear if replicative senescence is (i) an artifact of cell culture, caused perhaps by unphysiological oxygen partial pressure; or (ii) if replicative senescence does occur *in vivo*, and, if yes, if it is causative for organismic aging (as opposed to cellular aging), and (iii) if it is related to the development of human (or mouse) cancers.

To clarify these questions, it was necessary to identify reliable and sufficiently specific biochemical markers of cellular senescence in order to find a tool for monitoring and influencing senescence. One of the earliest markers found that was also believed to be the major cause for aging is telomere shortening (23). Other useful biochemical markers were identified in the form of loss of lamin B1, which is implicated in structural changes of the nucleus with senescence (24), as well as senescence-associated beta-galactosidase (SA β-GAL) (6). The present view is that the increase in SA-β-GAL is an indication of the proliferation of the lytic compartment in senescent cells due to increase of GLB1 (25). Therefore, this marker is not entirely specific for the process. Recently, staining of cells and tissues by Sudan Black B was introduced as novel marker for cellular senescence that is also applicable to paraffin sections. Sudan Black B stains lipofuscin, which are aggregates of oxidized proteins, lipids, and metals (15, 17). Interestingly, this staining method appears to be specific for cellular senescence, although lipofuscin would also be expected to accumulate in non-senescent cells during chronological aging.

An upcoming, highly promising method for the label-free detection of senescent cells *in vitro* and *in situ* is vibrational (micro)spectroscopy. Indeed, first proof of principle for Raman- and near-infrared spectroscopy, followed by multivariate statistics has been achieved as it was able to distinguish different cell types and cellular states in a non-invasive manner. First results on different human fibroblast strains, which were cultivated in 2D and 3D and subjected to serial passaging to induce replicative senescence, are very promising and allowed classification of cells at high confidence (26, 27). However, it needs to be determined if these methods are also applicable to other cell types, as well as to other inducers of cellular senescence. In the future, vibrational spectroscopy might allow to distinguish *in vivo* and in real time different cell types within the tumor stroma (28, 29), such as cancer cells, normal epithelial cells, and different subtypes of CAF, as well as determine how these cells respond to therapy by induction of either senescence or apoptosis (30).

Presently, expression of p16^INK4A^, one of the protein inhibitors of the cell cycle regulator cyclin dependent kinase (CDK), is the most reliable senescence marker known (14). While the senescence response also requires the main tumor suppressor proteins p53 and retinoblastoma protein (Rb), p16^INK4A^ seems to be expressed exclusively in senescent cells. Reporter constructs based on the promoter of p16^INK4A^ have successfully been used to detect senescent cells *in vivo*, but more importantly, also to test the physiological relevance of senescent cells in organismic aging (31, 32). Interestingly, p16^INK4A^ shares the same gene locus with two other important proteins involved in senescence and cancer.

### The INK/ARF Locus—At the Crossroads between Cancer and Senescence

The potent tumor suppressor proteins p16^INK4A^, p15^INK4B^, and p14^ARF^ are all transcribed from the same gene locus and are frequently targeted for deletion or epigenetic inhibition in numerous cancers. Although p16^INK4A^ and p14^ARF^ are both able to arrest the cell cycle and share exons 2 and 3, they comprise different amino acids and thus exert different biological functions due to alternative reading frames. Mouse models lacking either p16^INK4A^, p19^ARF^, which is the mouse homolog of human p14^ARF^, or both, always show increased incidence of various tumors, while human cancers frequently display either deletion of the whole gene locus, affecting both alternative reading frames, or specific silencing of either the p16^INK4A^ or p14^ARF^ promoter by methylation (33).

p16^INK4A^ and p15^INK4B^ bind to CDK4 and CDK6 and thereby promote allosteric changes, which inhibit CDK4/6-mediated phosphorylation of Rb. Thus, expression of p16^INK4A^ and p15^INK4B^ maintains Rb in a hypophosporylated state, which induces G1 cell cycle arrest (34). p14^ARF^ on the other hand stabilizes p53, the other main cellular tumor suppressor besides Rb, by trapping MDM2 in the nucleolus. This leads to increased p21 transcription and consequently to cell cycle arrest. However, p14^ARF^ can also act in a p53-independent manner by interaction with numerous other target proteins (33, 34).

Although different inducers of cellular senescence seem to converge on p16^INK4A^ in most cell types, and p14^ARF^ might or might not be co-regulated depending on the tissue context, the precise individual contributions of these pathways to the senescent state are not resolved yet.

### Replicative Senescence

The induction of cellular senescence was for a long time attributed to telomere shortening alone, for instance by serial passaging of cell cultures (23). Multiple cell divisions cause the telomeres to shorten to a critical length, which activates a persistent DNA damage response, leading to an upregulation of growth-inhibitory genes, such as p16^INK4A^ and p53, and repression of genes promoting the progression of the cell cycle (35). Before also other inducers of cellular senescence were discovered, the contribution of replicative senescence to aging *in vivo* was heavily debated.

One of the main reasons for this was the lack of suitable model systems, as 2D cell cultures alone hardly reflect the physiological state of an organism and mice have exceptionally long telomeres that complicate the interpretation of replicative senescence contribution. For instance, mice lacking TERC, the RNA component of telomerase, show progressive telomere shortening with age, although this does not manifest in any phenotype in the F1 and F2 generation. Clearly, this suggests that in wild-type mice (with long telomers) telomere shortening does not contribute to organismic aging. Only in the F3 generation, when the telomeres shortened to a critical length, a partial progeroid phenotype appears, which encompasses increased incidence of neoplasia. This phenotype is further pronounced in the F6 generation, when mice are in addition already short lived and sterile (36). Although these mouse studies indicate a contribution of telomere shortening to organismal aging and appearance of aging-associated neoplasia, in humans only few studies could correlate telomere length with longevity and improved health at old age (37–39). In numerous other studies, telomere length of various cell types including blood leukocytes was not found to be a reliable predictor of biological age and mortality (38).

Similarly, although the accumulation of short telomeres with age is expected to be associated with genomic instability and thus also with increased cancer incidence (39), this is not always the case in humans. On the contrary, some individuals with constitutively long telomeres in somatic cells show an increased propensity of major cancers at increasing age, while many cancer cells have short telomeres. Aviv and coauthors recently proposed a Two-Hit-Hypothesis that resolves this “Telomere Length Paradox” as follows: the first hit of mutations leading to cancer happens at stem cell level is, therefore, telomere length independent and leads to the expansion of fast-growing clones. Then, additional hits that are telomere length dependent and might occur much later in life induce the transformation of these expanding, but still benign clones into cancer. Thus, cells from individuals with constitutively long telomeres have a much longer expansion phase before entering cellular senescence and thereby suffer from an increased hazard of acquiring a second hit required for malignant transformation (40). Although this model is indeed able to explain many aspects of the correlation of telomere length with cancer incidence, experimental evidence is still lacking and probably hard to obtain due to difficulties with standardized absolute measurements of telomere length across different labs.

### Stress-Induced Premature Senescence (SIPS) and Therapy-Induced Senescence (TIS)

**Figure 1:**
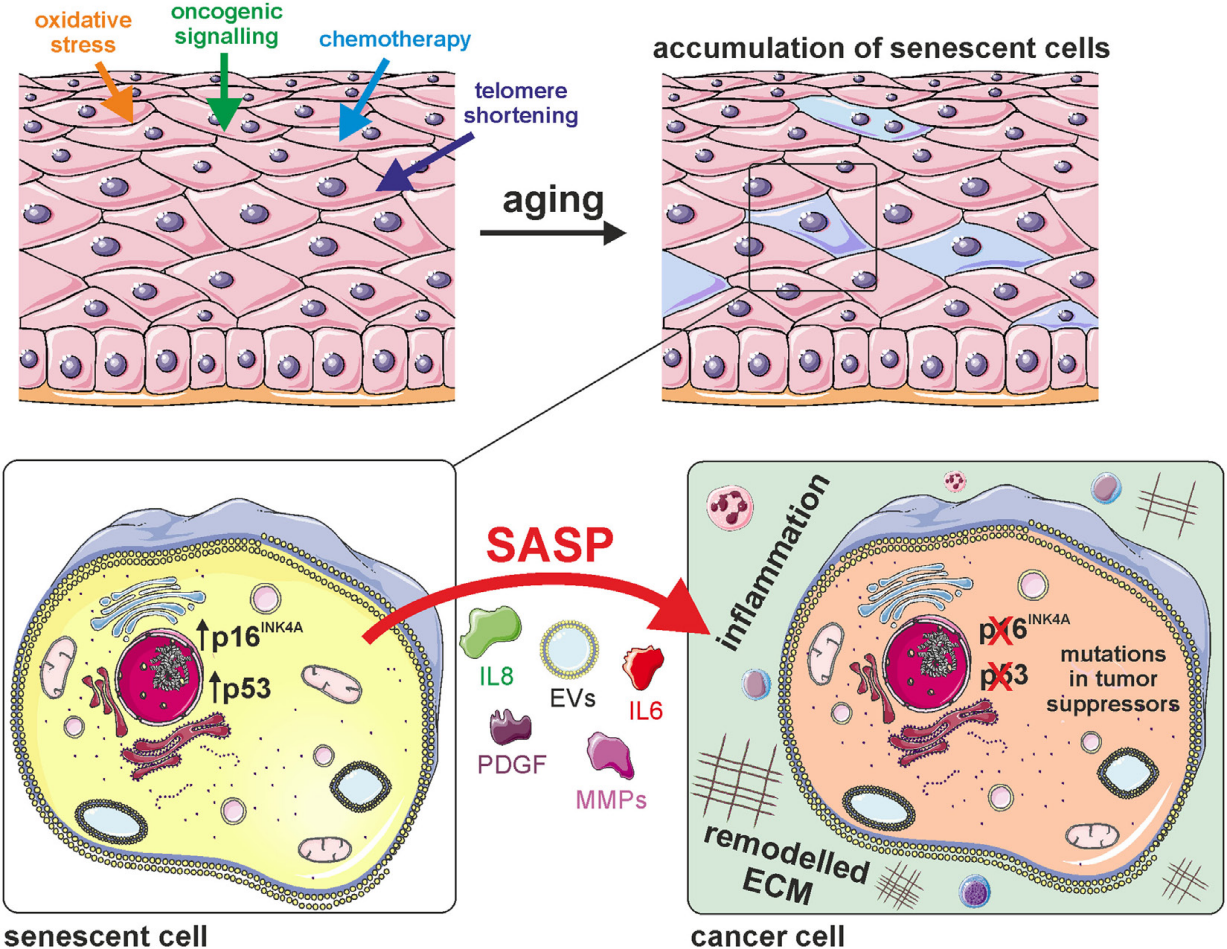
Cellular senescence generates a pro-tumorigenic microenvironment. Cellular senescence is induced by various stimuli that lead to the accumulation of senescent cells in aged tissues. The senescent state is characterized by activation of the potent tumor suppressors p16^INK4A^ and/or p53, as well as by secretion of various cytokines (e.g., IL-6, IL-8), growth factors [e.g., platelet-derived growth factor (PDGF)], matrix-metalloproteinases (MMPs), and extracellular vesicles (EVs). This senescence-associated secretory phenotype (SASP) generates a pro-tumorigenic microenvironment by inducing extracellular matrix remodeling and inflammation.

Besides the shortening of telomeres, senescence can also be induced by exposure of cells to acute or chronic sublethal doses of exogenous or endogenous stressors (Figure 1), causing a state of “stress-induced premature senescence” (41), “stress or aberrant signaling-induced senescence,” (35) or “accelerated cellular senescence” (42). Irrespective of the inducer, SIPS-cells are irreversibly growth arrested and express typical senescence markers, including SA β-GAL, p16^INK4A^, and telomere-associated persistent DNA damage foci (43). SIPS is likely the most important inducer of cellular senescence *in vivo*, since many cell types never exhaust their maximum replicative potential during organismal life span and thus do not enter replicative senescence, but are nevertheless exposed to various exogenous and endogenous stressors throughout life, which include ROS produced by the cell itself, cytotoxic compounds from the environment, radiation, or others.

Of high importance in the context of this review is the fact that many cytotoxic compounds as well as high dose radiation, which are currently used in cancer therapy to induce cell death, are also able to initiate senescence. Induction of senescence in cancer cells is often intended, as lower doses than for the induction of cell death are required and immediate severe side effects of therapy are minimized (44). These side effects include immunosuppression, fatigue, anemia, nausea, diarrhea, and alopecia (45). Furthermore, even cancer cells deficient in apoptosis pathways or lacking p53 and Rb retain their ability to undergo cellular senescence, rendering them sensitive to chemotherapy (46). This specific and important form of SIPS is referred to as “therapy-induced senescence.” Typical cytotoxic drugs in clinical use that induce DNA damage and thereby cellular senescence encompass Bleomycin, Camptothecin, Cisplatin, Doxorubicin, and Etoposide among others, with Doxorubicin and Cisplatin being most effective in initiating the senescence response. Biopsies from breast and lung tumors confirm that senescent cancer cells are indeed present *in vivo* in response to chemotherapy (42, 44, 47).

While induction of cellular senescence in tumor cells is beneficial for the therapeutic outcome, treatment-induced bystander senescence in other cells of the tumor stroma or even in distant tissues is not intended, as senescent cells might promote tumor relapse, secondary tumors, and tissue degeneration (35, 45). Demaria and coworkers could clearly establish that Doxorubicin and Paclitaxel induce senescence in normal mouse and human fibroblasts *in vitro*. Furthermore, systemic administration of either doxorubicin, paclitaxel, cisplatin, or temozolomide in mice induced cellular senescence *in vivo* in different cell types of skin, lung, and liver (45).

### Oncogene-Induced Senescence (OIS)

Oncogene activation and chemotherapy also induce premature senescence that is similar to replicative senescence regarding cell morphology and the expression profile of molecular markers (Figure 1). OIS was discovered by Serrano and coworkers more than 20 years ago (48, 49). It came as a surprise, because the paper essentially showed that the same dominant point mutation, which was found in many human tumors (H-rasV12) and was shown to be causative for cancer growth in combination with other mutations (for instance in the gene myc), was in isolation causing cell cycle arrest and cellular senescence, a nearly opposite phenotype compared to cancer growth. 20 years after this seminal discovery, we today see a bit more clearly the biological role of senescence in cancer biology. The above-mentioned point mutation in H-rasV12 or the corresponding mutation in yeast (RAS2-ala18, val19) cause a massive increase in ROS, which are inferred to transmit a signal causing senescence in the human case, and loss of growth regulation and subsequently apoptotic cell death in yeast (50, 51).

Oncogene-induced senescence does occur not only in cell culture, but also in tumors *in vivo* (52). Senescent cells in tumors are detected mostly in early pre-invasive stages of the tumor, but in later invasive stages are no longer detectable (53). OIS leads to SIPS *in vivo* and can induce tumorous growth in surrounding stroma cells. However, the molecular markers of OIS and the composition of the SASP depend on the experimental conditions and *in vivo* on the exact type of cancer. In one example (54), this included expression of the stem cell marker CD34^+^ in skin cancers derived from keratinocytes in the mouse model.

Taken together, the results available to date indicate that senescent cells produced by OIS in tumors can be both growth inhibiting and, in the long term, cancer causing. Many open questions remain, for instance: is the senescent state in tumor cells reversible *in vivo*? What are the phenotypic differences between OIS in tumors and senescence in development and regeneration? What is the mechanistic cause leading cells to choose senescence rather than apoptosis in OIS? What are the reasons and consequences of the strong pro-inflammatory phenotype of senescent cells in tumors?

## Senescent Cells Generate a Pro-Tumorigenic Microenvironment

### The Composition of the SASP

We have to acknowledge the fact that cellular senescence in certain cancers *in vivo* and in cancer-derived cell cultures *in vitro* can on the one hand exert an anticancer activity, because senescence is a permanent cell cycle arrest, and on the other hand, the secretion of various cytokines and chemokines by senescent cells induces de-differentiation and consequently increased cell division and even metastasis in neighboring cells (21, 22, 55, 56). This phenomenon is dubbed SASP and is studied in a large number of experimental systems, including not only senescent cells in old individuals and in tumors but also in wound healing and in embryogenesis (9–11). In the large majority of cases studied, the SASP does occur and the secreted soluble factors comprise interleukins, inflammatory cytokines, and growth factors. Interleukin-1 (IL-1) and interleukin-6 (IL-6) are expressed by senescent epithelial cells, fibroblasts, and other cell types and can induce either cellular senescence or tumor formation in neighboring cells. Chemokines secreted by senescent cells encompass interleukin-8 (IL-8), MCP-1, -2, -3, and -4, HCC-4, eotaxin-3, and MIP-1α and -3α (21). Interestingly, OIS cells secrete a range of CXCR-2-binding chemokines, which reinforce the senescent arrest in an autocrine manner (57). The SASP is also enriched for almost all IGF-binding proteins and their regulatory factors, which can induce senescence and apoptosis in neighboring cells (21, 58), as well as platelet-derived growth factors (PDGF) and vascular endothelial growth factors promoting wound healing. PDGF-A was shown to be enriched in the secretome of senescent mouse embryonic fibroblasts and to promote myofibroblast differentiation. As a consequence, clearance of senescent cells in the p16-3MR mouse model retarded the closure of wounds (11). In this mouse model, the p16^INK4A^ promoter, which is specific for senescent cells, drives both expression of *Renilla* luciferase and herpes simplex thymidine kinase (TK). By addition of gancyclovir, a suicide substrate of TK, only those cells are killed which activate the p16^INK4A^ promoter (11, 32).

In addition to soluble signaling factors, the SASP comprises proteases of the matrix metalloproteinase and serine protease family, which facilitate tissue repair by degradation of collagen and regulate the activity of other SASP factors (21, 59). Furthermore, the large insoluble glycoprotein fibronectin is preferentially transcribed and secreted by senescent cells. Fibronectin interacts with various other macromolecules, such as components of the cytoskeleton, cell surface receptors, and extracellular matrix components and thereby modulates processes such as cell adhesion and proliferation (60, 61).

Although not as well characterized as soluble proteins, also other macromolecules such as lipids and carbohydrates, as well as nucleic acids and proteins enclosed in extracellular vesicles (EVs), are SASP members. Senescent cells secrete increased amounts of small extracellular vesicles (sEVs) that promote proliferation of cancer cells and exert other effects on bystander cells (62, 63). The mode of action is partially attributed to EphA2, which is phosphorylated upon cellular senescence and specifically packaged into sEVs. Together with Ephrin-1, which is expressed by cancer cells, reverse signaling *via* Erk is initiated and proliferation of cancer cells is stimulated (62). EVs also contain miRNAs that are able to exert paracrine effects on gene expression of other cells. Since miRNA expression patterns differ significantly between senescent and non-senescent cells (64), miRNAs will probably soon be recognized as novel and important SASP members.

Importantly, the composition of the SASP significantly varies from cell type to cell type and thereby might differently influence bystander cells (44, 59).

### Actions of the SASP on Surrounding Cells and the Extracellular Matrix

Effects of the SASP on surrounding cells strictly depend on the tissue context. In most cases the SASP was reported to stimulate tumor growth (Figure 1), but on the other hand immune cells are attracted which participate in the clearance of cancer cells (65). Another example for context specific roles of senescent cells is liver cancer: oncogene-induced senescent hepatocytes secrete CCL2, which attracts CCR2^+^ myeloid cells that further differentiate into macrophages and clear pre-malignant cells. If, however, hepatocellular carcinoma is already established, cancer cells block the maturation of the attracted CCR2^+^ myeloid cells into macrophages and thereby also inhibit NK cells. In this scenario, the presence of senescent cells promotes tumor outgrowth and thus worsens the prognosis for patients (66).

Despite its role in inducing bystander senescence, the SASP has also an important function in tissue plasticity and stemness. Ritschka and coworkers demonstrated that the SASP promotes the expression of stem cell markers *in vitro* and *in vivo* and transient exposure to the SASP induces stem cell functions. Chronic exposure, however, had an opposite effect, probably due to paracrine senescence induction in stem- and progenitor cells (54). The close relationship between senescence and tissue regeneration is further emphasized by a mouse model of ectopic expression of the transcription factors OCT4, SOX2, KLF4, and cMYC (OSKM), which are required for the induction of pluripotency. In these mice, tissues harboring a high proportion of senescent cells also displayed a high *in vivo* reprogramming efficiency and *vice versa*. Mosteiro and coworkers identified IL-6 as critical SASP factor for reprogramming, as well as tissue damage as a possible inducer (67).

The importance of typical SASP factors for tissue regeneration and wound healing might explain their evolutionary conservation and, in addition, SASP factors promote epithelial to mesenchymal transition, a hallmark during the development of carcinomas and angiogenesis (21). Therefore, we propose that not only senescence itself is antagonistically pleiotropic but also the corresponding SASP, as it might be beneficial in young individuals for wound healing and tissue regeneration, while tumor promoting in the elderly.

### The Extracellular Matrix Is an Important Contributor to a Pro-Carcinogenic Microenvironment

Remodeling of the extracellular matrix by metalloproteinases of the SASP might create a beneficial microenvironment for tumor growth, as migration is facilitated and contact inhibition is blunted (Figure 1).

An interesting model organism to study the development of pro- and antitumorigenic microenvironments by modulation of the extracellular matrix is the naked mole rat. These animals are exceptionally long lived and suppress the development of cancer by expression of high molecular mass hyaluronic acid that renders the extracellular matrix highly viscous and thereby cells become extremely sensitive to contact inhibition (68). Interestingly, this phenomenon is associated with elevated expression of p16^INK4A^ (69), and naked mole rat fibroblasts are more tolerant to cellular stress than mouse fibroblasts, because they halt cell proliferation at much lower doses of stressors (70). Thus, a denser extracellular matrix might promote increased expression of p16^INK4A^, which allows cells to sense lower doses of toxic compounds and consequently enter a state of cell cycle arrest.

In order to further interrogate the complex relationship between cellular senescence, cancer formation, and the extracellular matrix, it would be very interesting to determine if this cell cycle arrest is irreversible and thereby resembles cellular senescence, as well as to test for presence of senescent cells in naked mole rats *in vivo*.

### Senescent Cells and CAF: United by a Similar Secretory Phenotype?

Cancer-associated fibroblasts are a heterogeneous population of fibroblasts within the tumor stroma that is only poorly characterized so far. Most of these cells originate from normal local fibroblasts, which are stimulated by members of the PDGF or TGF-β family, but also normal endothelial or epithelial cells that underwent epidermal to mesenchymal transition, as well as bone-marrow derived mesenchymal stem cells contribute to the CAF population. Tumor cells release paracrine factors that attract CAF, support their survival within the tumor microenvironment, and stimulate their secretory phenotype. In contrast to normal fibroblasts, CAF express more factors associated with degradation of the ECM and increased angiogenesis, but also chemokines promoting tumor cell proliferation, migration, and invasion (1).

Two prominent sub-populations within CAF, namely senescent fibroblasts and myofibroblasts, both express α-smooth muscle actin and promote tumor cell mobility and thereby malignancy by secretion of soluble factors. Gene expression profiles between the two CAF subtypes differ, with myofibroblasts and non-senescent cells stimulating collagenous ECM deposition and thereby causing poor prognosis (71).

The tumor-promoting effects of CAF are mainly attributed to CXCL12, which is expressed and secreted by CAF (72), but is also an important SASP component (73). CXCL12 induces tumor proliferation, angiogenesis at the tumor site, and invasion, leading *in vivo* to increased tumor development and metastasis (1). Other tumor-promoting chemokines, which are secreted by CAF as well as by senescent cells, are SDF-1, GROα, GROβ, IL-8, MCP-1, and MCP-8 (1, 21, 74). miR-335 is upregulated in both CAF and normal senescent fibroblasts and is able to modulate secretion patterns of both cell types (75).

**Figure 2:**
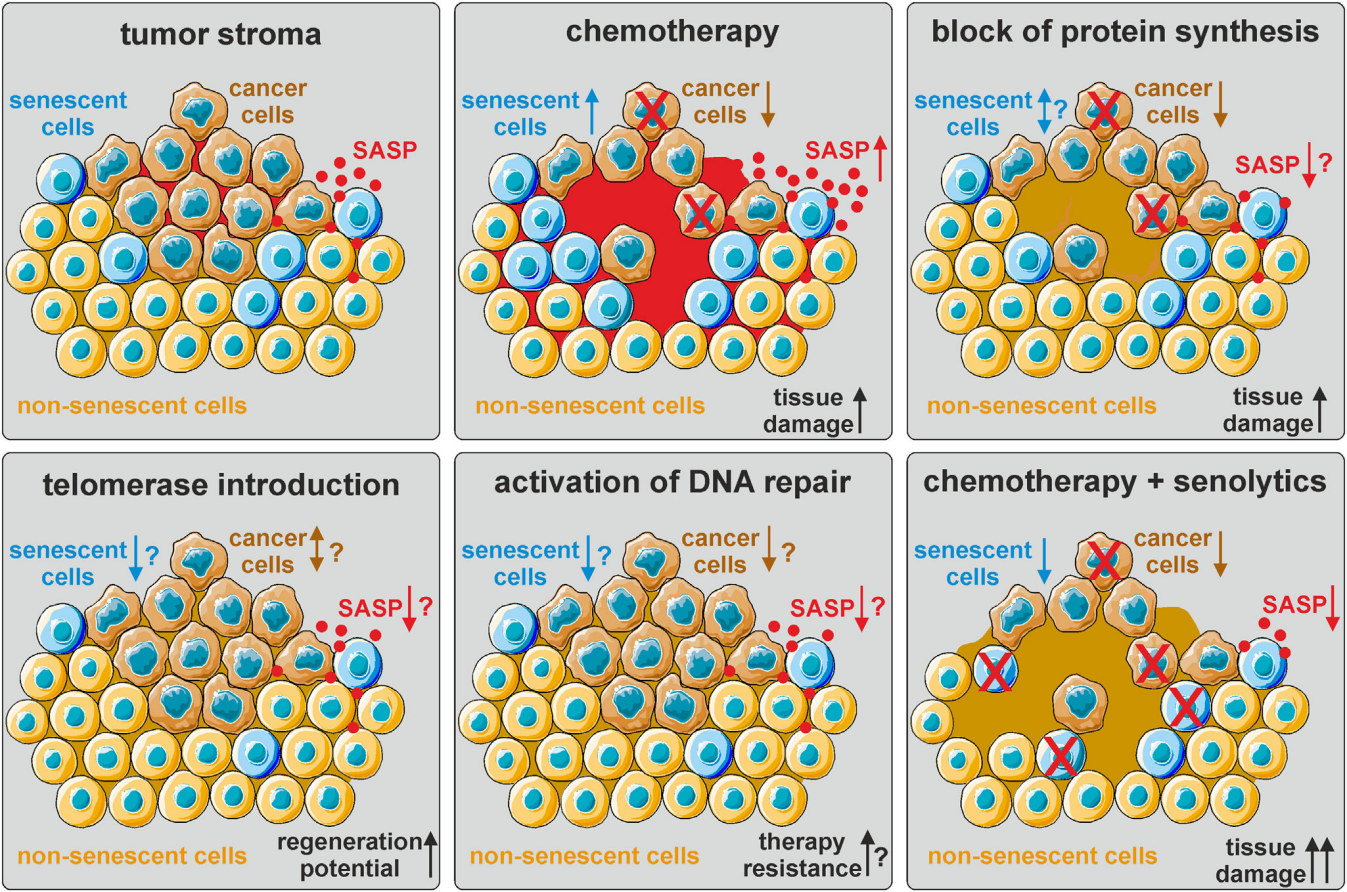
The influence of current and hypothetical therapy options on the tumor stroma. The tumor stroma is comprised of tumor cells (brown), normal non-senescent cells (orange), and senescent cells (blue), as well as other cell types (not depicted here). The senescence-associated secretory phenotype (SASP) generates a pro-tumorigenic microenvironment (red). Chemotherapy eliminates cancer cells by inducing DNA damage, but also induces cellular senescence and thereby promotes secondary tumor formation and relapse *via* SASP upregulation. Block of protein synthesis also eliminates cancer cells, but might additionally mitigate the SASP. The effect on the number of senescent cells is not known. Telomerase (re-)introduction in normal cells might delay the onset of cellular senescence and promote tissue regeneration, but also facilitate cancer development. Activation of DNA repair pathways in non-senescent cells might prevent senescence and cancer formation, but also render cancer cells more resistant to chemo- and radiation therapy. Thus, a combinatorial approach of eliminating cancer cells by chemotherapy and senescent cells by senolytics might be most promising. However, increased tissue damage combined with decreased regenerative ability by SASP factors needs to be considered.

Taken together, these data clearly indicate similar secretory phenotypes and underlying regulatory networks of CAF and senescent cells, which generate a pro-tumorigenic microenvironment. Thus, senescent cells can serve as an important *in vitro* model for microenvironments favoring tumor growth. This is especially relevant for the evaluation of current cancer therapies, which mostly rely on cytotoxic compounds and/or radiation that drive cancer cells into either apoptosis or TIS. However, senescence is also induced in non-cancer cells, which further promotes the SASP and thereby exacerbates deleterious side effects (Figure 2).

## Upcoming Innovative Therapeutic Approaches

### Targeting Cancer and Senescence Simultaneously by Modulation of Protein Synthesis

Protein synthesis is highly upregulated in cancer in order to support fast tumor growth (12) and is conducted by ribosomes, which are complex nanomachines assembled in the nucleolus. Ribosome biogenesis and consequently nucleolar size are directly correlated with cell cycle and protein synthesis. Thus, the morphology of nucleoli serves as an important surrogate marker for tumor pathologists to predict the clinical outcome of cancer (76). In contrast to cancer, bulk protein synthesis slows down during organismal aging (77). Although it is already established that senescent cells display decreased levels of protein degradation (78), the capacity of senescent cells to newly synthesize proteins was not studied so far. Surprisingly, primary fibroblasts from Hutchinson–Gilford Progeria patients and from old donors display elevated protein translation and nucleolar expansion compared to fibroblasts from healthy young donors (79), while long-lived *Caenorhabditis elegans* mutants have smaller nucleoli and less ribosomal RNA expression than their wild-type counterparts (80). Replicative senescent fibroblasts are characterized by a single enlarged nucleolus, while proliferating cells have an increased number of small nucleoli (81–83). However, although senescent cells undergo vast nuclear remodeling (18, 19), the relative positions of nucleolus-associated chromosomal domains only change marginally during senescence (84). Interestingly, the nuclear proteome is drastically remodeled in SIPS compared to untreated proliferating cells with an accumulation of ribosomal proteins and depletion of ribosome biogenesis factors. Taken together, these findings indicate a kinetic shift of ribosome assembly in senescent cells (85) and firmly establish that increased ribosome and protein syntheses, as well as nucleolar expansion, are hallmarks of both cancer and accelerated aging.

Thus, therapies targeting protein synthesis instead of inducing generalized DNA damage are supposed to be more selective for cancer cells, avoid the induction of senescence in non-cancer cells, and slow down organismal aging and aging-associated pathologies (Figure 2). Indeed, inhibition of ribosome biogenesis, for instance by RNA polymerase I inhibitors, selectively kills cancer cells and is thereby considered to be a promising novel therapeutic option (86).

Another approach, which could simultaneously target tumor progression and organismal aging, is the inhibition of mTOR by Rapamycin or Rapamycin-analogs as inhibitors of protein synthesis. Rapamycin, even if administered late in life, extends organismal life span in mice (87) and blocks secretion of pro-tumorigenic components of the SASP, such as IL-6. This effect is achieved by specific translational repression of IL-6, as well as on transcription level *via* a feedback loop involving IL1A and NF-κB signaling. Importantly, Rapamycin also reduced tumor formation in a xenograft model *in vivo* (88). Thus, it would be very interesting to evaluate if other drugs that inhibit protein synthesis are also able to limit cancer progression while mitigating negative paracrine effects of senescent cells. Potential drug targets include several nucleolar proteins required for ribosome biogenesis.

One of these factors is Nucleophosmin 1 (B23), which is upregulated in adenomas and cancers of the colon (89) and translocates from the nucleolus to the nucleoplasm upon SIPS. B23 gene silencing by RNAi induces reductions in cell viability as well as increased abundance of senescent cells. Knockdown of p53 rescues this phenotype, indicating that p53 is required for B23-knockdown-mediated senescence (85).

Another remarkable nucleolar factor orchestrating the nucleolar stress response and linking cellular senescence and cancer initiation is nucleomethylin (NML). NML introduces specific m^1^A 28S ribosomal RNA methylation in human and mouse cells and contributes to 60S subunit formation. Depletion of NML activates the p53 pathway and thereby regulates cellular proliferation (90). Interestingly, NML is important for the induction of drug-induced senescence in tumor cells. Upon depletion of NML, the probability of cells to escape senescence increases, which might cause relapse after chemotherapy. Indeed, downregulation of NML correlates with poor survival of breast cancer cell patients (91).

A very recent report describes NOLC1, which is a nucleolar protein with increased expression in cellular senescence and decreased expression in liver cancer. Strikingly, NOLC1 overexpression promotes the onset of senescence and represses hepatocellular carcinoma proliferation, both *in vitro* and in xenograft models. NOLC1 overexpression decreases rRNA synthesis and alters the morphology of nucleoli toward ring-like structures (92).

Taken together, these studies demonstrate that more and more genes are emerging, which establish a clear link between nucleolar stress, cellular senescence, and cancer, although most mechanistic details of these pathways remain elusive. Still, ribosome biogenesis and protein synthesis are important novel drug targets allowing decoupling of cancer therapy and bystander senescence induction.

### Strategies to Decrease Cellular Senescence and Thereby Cancer

#### Telomerase—A Two-Edged Sword that Delays Senescence but Promotes Cancer

Telomerase is a reverse transcriptase that is mainly expressed in germ-, stem-, and cancer cells and counteracts telomere shortening induced by multiple cell divisions. Ectopic overexpression of telomerase is able to immortalize a wide range of different cell types (93–95). Although re-activation of telomerase is in many cases one of the critical steps in carcinogenesis, activation of telomerase by pharmacological compounds or gene therapy is still considered a promising strategy to promote tissue regeneration and delay various senescence-associated pathologies.

Several studies have already firmly established the dual role of telomerase in cancer and aging. Ectopic overexpression of TERT, the catalytic subunit of telomerase, promotes both tumor formation (96) and longevity in the mouse (97, 98). The increase in longevity is only observed in a tumor-resistant genetic background (97) or if telomerase expression is initiated late in life by gene therapy. In this scenario, TERT extends life span in 1- and 2-year-old mice, blunts the age-dependent loss of adipose tissue mass, bone density and coordination, but does not increase cancer incidence (98). The authors argue that the tumorigenic activity of telomerase is strongly repressed in aged organisms.

Nonetheless, pharmacological or gene therapy-mediated activation of telomerase to ameliorate aging-associated pathologies, including several forms of cancer, is heavily debated in the field. One side argues that re-expression of telomerase in old organisms, in synergy with caloric restriction (99) or in non-proliferative tissues, such as the heart, is not only safe, but also promotes tissue regeneration, such as after myocardial infarction (100). Others believe that expression of telomerase still poses the risk of increased cancer incidence by loss of a main tumor suppressor checkpoint (Figure 2). The role of telomerase in cancer and senescence was already extensively reviewed elsewhere (101).

#### Promotion of DNA Damage Repair to Reduce Senescence and Cancer Incidence

Apart from the activation of telomerase, a few other interventions were described in literature that are able to postpone the onset of cellular senescence and increase stress resistance in experimental models (Figure 2).

Our group could demonstrate that ectopic overexpression of SNEV^Prp19/Pso4^ extends the replicative life span of human endothelial cells (102, 103), as well as the organismal life span of fruit flies (104). Although SNEV^Prp19/Pso4^ participates also in pre-mRNA splicing (105) and in the ubiquitin/proteasome-pathway (106), we believe that its involvement in DNA damage repair is responsible for the increased stress resistance and fitness. Indeed, SNEV^Prp19/Pso4^ and other DNA repair factors are also partially required for adipogenic differentiation of human adipose stromal cells and fat accumulation in *Caenorhabditis elegans* (107). Thus, enhancement of DNA damage repair, for instance by (not yet existing) small molecules activating SNEV^Prp19/Pso4^, could potentially mitigate the accumulation of senescent cells upon aging or cancer therapy with cytotoxic compounds. Interestingly, SNEV^Prp19/Pso4^ expression is elevated in breast cancer cells, but tumors with high SNEV^Prp19/Pso4^ levels display reduced metastatic potential (102). Thus, animal experiments are required in order to evaluate benefits and detrimental effects of SNEV^Prp19/Pso4^ overexpression regarding cancer incidence, metastasis, life span, and fitness at old age. In addition, it should be considered that improved DNA repair capacity in tumor cells is expected to reduce the efficacy of chemo- and radiation therapy.

Another promising approach that is already in clinical trials for the treatment of lung cancer is introduction of p53 by gene therapy (108). Thereby, cancer cells are sensitized for chemo- and radiation therapy. Since mouse experiments suggest that p53 overexpression (Super-p53 mouse) protects from cancer but does not shorten life span (109), it would be very interesting to evaluate if p53 gene therapy increases numbers of senescent cancer and non-cancer cells in human biopsies.

The problem with these hypothetical therapeutic interventions is the fact that activation of certain pathways or genes is usually more difficult to achieve by pharmaceuticals than their inactivation. Furthermore, it is very hard to predict how compounds targeting cancer affect senescence and *vice versa*, as pathways are tightly intertwined. Thus, selective clearance of senescent cells is in our view currently the most promising strategy to counteract aging- and therapy-associated senescence.

### Elimination of Senescent Cells Mitigates Side Effects of Cancer Therapy

#### Genetic Clearance of Senescent Cells

The idea is to selectively kill senescent cells and to analyze if this can lead to rejuvenation of the organism, recovery from typical diseases of old age, which are believed to be caused by an accumulation of senescent cells, or recovery from the aging phenotypes which are caused by chemotherapy in cancer patients (44, 110). Basically, two approaches, namely, genetic clearance of senescent cells and senolytic compounds have been developed over the last years and both have proved to be highly successful in the mouse model (7, 11, 31, 32), but have not yet reached clinical application.

Genetic clearance of senescent cells relies on the genomic integration of either the *INC-ATTAC* (7, 31) or p16-3MR (11, 32) reporter construct, which were developed independently by two different labs, and enable recognition of p16^INK4A^ -expressing cells in senescence accelerated mice, but also in naturally aged mice, both at cellular and organismic level. In addition, both constructs allow conditional induction of apoptosis specifically in p16^INK4A^ positive cells upon systemic administration of either AP20187 (for *INC-ATTAC*) or ganciclovir (for p16-3MR). Importantly, clearance of senescent cells by *INC-ATTAC* increased life span, but did not decrease tumor incidence. However, mice having a tumor at the time of death showed increased survival (7). Genetic clearance of senescent cells in p16-3MR mice that were systemically treated with Doxorubicin was able to mitigate side effects of that drug, such as cardiac dysfunction, generalized increase of inflammation, and loss of hematopoetic stem cell function. In addition, genetic clearance of senescent cells reduced the incidence of cancer relapse and metastasis, as well as fatigue after Doxorubicin treatment (45). Thus, clearance of senescent cells might have clinical potential to reduce short-term and long-term side effects of current cancer therapy.

The downside of this approach is the fact that genetic clearance of senescent cells is not equally efficient in all organs (7, 31) and senescent cells lacking p16^INK4A^ cannot be targeted. Thus, it would be very interesting to compare the current mouse models to other *in vivo* models that rely on different senescence markers. However, these models do not yet exist.

#### Senolytics—Translation of Genetic Mouse Models into Clinical Application

With the knowledge that specific elimination of senescent cells is able to slow organismal aging, several groups underwent the endeavor to screen for substances that specifically kill cells that are senescent, but not proliferating or reversibly growth arrested. Dasatinib and quercetin were identified as one of the first “senolytic” compounds and evaluated in the *INC-ATTAC* system *in vivo* (111). The results show that senescent cells are indeed eliminated, but the senolytic drugs which are presently known are not sufficiently specific, they also attack non-senescent cells (111, 112).

Therefore, an interesting strategy to specifically target senescent cells was introduced by Doerr and coworkers. The authors found that TIS-induced lymphoma cells have a much higher glucose- and energy demand, as well as elevated proteotoxic stress than non-senescent cells or TIS-cells that do not produce SASP (113). Mitochondria are required to fulfill the high energy demands of senescent cells and, indeed, depletion of mitochondria was able to reduce senescence phenotypes including the SASP *in vitro* and even prevent the onset of senescence in mouse livers *in vivo* (114). Thus, this specific metabolic condition of senescent cells, which is maintained by mitochondria, could be exploited to design a novel class of senolytics.

Another new approach was recently presented based on a cell penetrating peptide, FOXO4-DRI. FOXO4 is a member of the FOXO (FOX other) family of transcription factors which is expressed exclusively in senescent cells and prevents cell death by binding to p53 thereby retaining p53 in the nucleus. The peptide, FOXO4-DRI (FOXO4 d-amino acids retro inverso) is modeled after a unique sequence in the interaction surface of FOXO4 with p53. It contains only d-amino acids and is, therefore, not degradable in the cell. Penetration into cells is afforded by fusion with a hydrophilic and basic short sequence of the HIV-TAT protein. The interaction surface of FOXO4 is exactly mimicked by the side chains of the d-amino acids and the peptide with high affinity prevents binding between FOXO4 and p53, which in a “natural” way leads to efficient apoptotic death of the cells after nuclear export and transfer to mitochondria of p53. The mouse experiments show that (i) in normally aged mice, kidney function is restored to near youthful levels (tested by plasma urea and creatinine), and the same is true for skin and fur phenotypes; (ii) in mice that were treated with doxorubicin and developed senescence phenotypes, especially liver damage, this disease phenotype was also repaired by the FOXO4-DRI peptide. The side effects monitored in the mice and in cultured cells were negligible when compared to other senolytic drugs (32).

In our view, these experimental results combined show now for the first time in senescence research that the accumulation of senescent cells is indeed causally contributing to at least some aging diseases and probably to aging in general. More importantly, in the context of the present review article, senescent cells in tumors and in tumor patients after chemotherapy can be treated by eliminating senescent cells, which is a very promising suggestion for the future of cancer therapy (Figure 2). However, it should be considered that elimination of senescent cells might impair tissue regeneration and, therefore, limit the repair of damage that was inflicted by chemotherapy. The correct timing, dosing, and patient selection by, e.g., specific companion diagnostics of senescent cell elimination and chemotherapy will be crucial in order to maximize therapy success and minimize side effects.

## Conclusion and Outlook

For a long time, cellular senescence was purely seen as *in vitro* phenomenon and its influence on human aging was very controversial in the field, until quite recently several groups could clearly establish that senescent cells indeed contribute to aging-associated diseases and ultimately to organismal life- and health span. In the last few years, senescence has come also more and more into focus in cancer research, as senescence is frequently induced by current tumor therapies, being beneficial for arresting apoptosis-resistant cancer cells, but on the other hand inducing senescence in other cells and thereby promoting cancer relapse and secondary tumors. In addition, the accumulation of senescent cells with age might at least partially explain increasing cancer incidence with age.

Since the secretory phenotypes of senescent cells and CAF are similar, cellular senescence serves as an interesting model system for a pro-tumorogenic microenvironment that could be utilized for drug screenings. Furthermore, pharmaceutically targeting senescent cells might not only be a novel tool in battling aging-associated pathologies, but also a complementation to cancer therapy to eliminate senescent cancer and non-cancer cells and mitigate side effects. The coming years will show if a better understanding of the complex interplay between cellular senescence and cancer will indeed revolutionize therapy options.

## Author Contributions

MS and MB wrote the manuscript. MS designed figures. JG provided ideas regarding the concept of the manuscript and critically revised the manuscript. All authors read, corrected, and approved the final version of the manuscript. MS prepared and submitted all required files.

## Conflict of Interest Statement

JG is cofounder and shareholder of Evercyte GmbH and TAmiRNA GmbH. All other authors declare no competing interests.
